# Severity and Patterns of Blood-Nerve Barrier Breakdown in Patients with Chronic Inflammatory Demyelinating Polyradiculoneuropathy: Correlations with Clinical Subtypes

**DOI:** 10.1371/journal.pone.0104205

**Published:** 2014-08-08

**Authors:** Fumitaka Shimizu, Setsu Sawai, Yasuteru Sano, Minako Beppu, Sonoko Misawa, Hideaki Nishihara, Michiaki Koga, Satoshi Kuwabara, Takashi Kanda

**Affiliations:** 1 Department of Neurology and Clinical Neuroscience, Yamaguchi University Graduate School of Medicine, Ube, Japan; 2 Department of Neurology, Graduate School of Medicine, Chiba University, Chiba, Japan; Hungarian Academy of Sciences, Hungary

## Abstract

**Objective:**

Chronic inflammatory demyelinating polyradiculoneuropathy (CIDP) is currently classified into clinical subtypes, including typical and atypical forms (multifocal acquired demyelinating sensory and motor neuropathy (MADSAM) and distal acquired demyelinating symmetric neuropathy (DADS)). The aim of this study was to elucidate the patterns and severity of breakdown of the blood-nerve barrier (BNB) in each CIDP subtype.

**Methods:**

We evaluated the effects of sera obtained from patients with typical CIDP, MADSAM and DADS and control subjects on the expression levels of tight junction proteins and transendothelial electrical resistance (TEER) value in human peripheral nerve microvascular endothelial cells (PnMECs).

**Results:**

The sera obtained from the patients with the three clinical phenotypes of CIDP decreased the amount of claudin-5 protein levels and TEER values in the PnMECs. In addition, the sera obtained from typical CIDP patients more prominently reduced claudin-5 protein levels and TEER values in the PnMECs than did that obtained from the MADSAM and DADS patients. Furthermore, the severity of BNB disruption after exposure to the sera was associated with higher Hughes grade, lower MRC score, more pronounced slowing of motor nerve conduction in the median nerve and higher frequency of abnormal temporal dispersion.

**Conclusions:**

Sera derived from typical CIDP patients destroy the BNB more severely than those from MADSAM or DADS patients. The extent of BNB disruption in the setting of CIDP is associated with clinical disability and demyelination in the nerve trunk. These observations may explain the phenotypical differences between CIDP subtypes.

## Introduction

Chronic inflammatory demyelinating polyradiculoneuropathy (CIDP) is a rare autoimmune-mediated neuropathy thought to constitute a group of heterogeneous disorders involving a wide range of clinical phenotypes, variable clinical course and differing responses to immunotherapy [1], [2]. The Joint Task Force of the European Federation of Neurological Societies and Peripheral Nerve Society (EFNS/PNS) convened in 2010 divided CIDP into two clinical subtypes: “typical CIDP (t-CIDP),” the classical pattern of CIDP, and “atypical CIDP,” which include multifocal acquired demyelinating sensory and motor neuropathy (MADSAM) and distal acquired demyelinating symmetric neuropathy (DADS) [3]. t-CIDP is clinically defined by the presence of chronically progressive or recurrent symmetrical proximal and distal weakness and sensory dysfunction in all extremities developing over at least two months and likely affects a relatively uniform group of patients [4], [5]. In contrast, MADSAM neuropathy is characterized by an asymmetrical multifocal pattern of motor and sensory impairment (mononeuropathy multiplex) likely representing an asymmetrical variant of CIDP [6], [7]. On the other hand, DADS neuropathy is characterized by symmetrical sensory and motor polyneuropathy of the distal upper and lower limbs predominantly associated with muscle weakness and/or sensory disturbances in the distal limbs [8], [9]. These three CIDP subtypes share a common feature, namely, chronic demyelinative neuropathy of supposed immune origin; however, the different clinical phenotypes appear to result from differences in the underlying immunopathogenesis [10].

Various previous reports have demonstrated that the pathological breakdown of the blood-nerve barrier (BNB), which allows for the entry of immunoglobulins, cytokines and chemokines into the peripheral nerve system (PNS) parenchyma, is a key event in the disease process of CIDP [11], [12], [13], and the result of electrophysiological examinations have led to a new hypothesis concerning the pathogenesis of CIDP, namely that differences in the degree of BNB malfunction partly determine the differences in both the distribution of demyelinative lesions and clinical phenotypes observed between t-CIDP and MADSAM neuropathy [10], . In the present study, we evaluated the contributions of humoral factors in sera obtained from patients with each clinical subtype of CIDP to BNB breakdown and clarified the association between BNB disruption and clinical profiles using our previously established human BNB-derived immortalized endothelial cells [16].

## Materials and methods

### Serum and cerebrospinal fluid samples

The study protocol was approved by the ethics committee of Yamaguchi University and Chiba University. All patients consented to participate and written informed consent was obtained from each subject. Serum was collected from a total of 25 CIDP patients with t-CIDP (n = 12), MADSAM (n = 10) and DADS (n = 3) in the initial progressive phase of the disease or at relapse, without either corticosteroid or intravenous immunoglobulin (IVIg) treatment, diagnosed at Chiba University Hospital or Yamaguchi University Hospital. All patients fulfilled the diagnostic criteria for CIDP based on the guidelines reported by the EFNS/PNS 2010 [3]. The inclusion criteria was a diagnosis of definitive or probable CIDP. None of the patients with DADS had anti-myelin-associated glycoprotein (MAG) antibodies. Sera obtained from 10 healthy individuals served as normal controls. All serum samples were inactivated at 56^○^C for 30 minutes just prior to use. Cerebrospinal fluid (CSF) samples obtained from the 25 patients with CIDP were analyzed with respect to the protein level in the CSF, the IgG index and/or CSF/serum albumin ratio (Q Alb). The clinical and electrophysiological data for all CIDP patients were analyzed. The clinical parameters included the Hughes functional grading scale [17], which was used as a functional assessment, and the total Medical Research Council (MRC) scale for four muscle groups (deltoid, wrist extensor, iliopsoas and tibialis anterior muscles). All 25 patients received immune system-modulating treatment, including corticosteroids and IVIg. Treatment was considered to be effective if the patient's condition, including the Hughes scale and MRC score, was found to have improved after therapy. Nerve conduction studies were performed according to conventional procedures and using standard electromyography machine (Neuropack M1, Nihon Kohden, Tokyo, Japan; Viking 4, Nicolet Biomedical Japan, Tokyo, Japan). Motor nerve studies of the median, ulnar and tibial nerves were performed, including F wave analyses. The terminal latency index (TLI) was calculated based on the following formula: TLI =  terminal distance (mm)/(distal latency (ms) × conduction velocity (m/s)). A partial motor conduction block was defined as a more than a 50% reduction in the compound muscle action potentials (CMAP) between the stimulus sites, and abnormal temporal dispersion was defined as a more than 30% increase in duration between the proximal and distal CMAP, in accordance with the EFNS/PNS guidelines [3].

### Cell culture and treatment

Immortalized human peripheral nerve microvascular endothelial cells (PnMECs), termed “FH-BNBs”, were generated previously in our laboratory [16]. The cells were cultured in medium [Dulbecco's modified Eagle's medium (DMEM; Sigma, St. Louis, MO, USA) containing 10% fetal bovine serum (FBS; Sigma, St. Louis, MO, U.S.A) and antibiotics] with 10% patient serum or culture medium containing 10% FBS, which was used as a control, in an incubator at 37^○^C with 5% CO_2_/air. The cells were maintained for either 24 hours to measure the transendothelial electrical resistance (TEER) value or 48 hours to extract total proteins.

### Reagents

We purchased polyclonal anti-claudin-5 and anti-occludin antibodies from Zymed (San Francisco, CA, U.S.A). Polyclonal anti-actin antibodies were purchased from Santa Cruz (Santa Cruz, CA, U.S.A).

### Western blot analysis

After boiling, aliquots containing equal amounts of protein (15 µg) were separated via SDS-PAGE (Bio-Rad, Hercules, CA). The proteins were then transferred onto nitrocellulose membranes (Amersham, Chalfont, UK), as previously described [18]. The membranes were subsequently treated with the relevant primary antibodies (dilution: 1∶100) for two hours and then incubated with the secondary antibodies (dilution: 1∶2,000) for one hour at room temperature. Finally, the proteins were visualized using an enhanced chemiluminescence detection system (ECL-prime, Amersham, UK). The optical density of each band was assessed using the Quantity One software program (Bio-Rad).

### Transendothelial electrical resistance (TEER) studies

The TEER values in the cell layers were measured using a Millicell electrical resistance apparatus (Endohm-6 and EVOM, World Precision Instruments, Sarasota, FL, U.S.A), according to the manufacturer's instructions. The cells were seeded (1×10^6^ cells/insert) on collagen-coated Transwell inserts (pore size: 0.4 µm, effective growth area: 0.3 cm^2^, BD Bioscience, Sparks, MD, USA), and the TEER value for each insert was calculated following treatment with each type of medium (non-conditioned medium was used as a control, the conditioned medium contained 10% patient serum) for 24 hours by subtracting the blank from each reading. Each condition was tested in triplicate for each experiment.

### Data analysis

Differences in the median values between the groups were examined according to the Mann-Whitney U test, with two-sided P value of <0.05 considered to be statistically significant. Pearson correlation coefficients were used to test the associations. All statistical analyses were performed using the IBM SPSS statistical software program, version 21J.

## Results

### Clinical characteristics

The clinical profiles of patients with t-CIDP, MADSAM and DADS are summarized in Table 1. The mean Hughes grade was significantly higher in the t-CIDP patients than in the MADSAM or DADS patients and in the MADSAM patients than in the DADS patients. In addition, significantly lower mean MRC values for both the total score for the four muscle groups and the iliopsoas alone were observed in the t-CIDP patients compared to those noted in the MADSAM and DADS patients. Meanwhile, the mean CSF protein concentration was higher in the t-CIDP and DADS patients than in the MADSAM patients. Based on the results of the electrophysiological examinations of the median nerve, the t-CIDP and DADS patients demonstrated a more prolonged average motor nerve distal latency than the MADSAM patients, and while the t-CIDP patients displayed greater slowing of mean motor nerve conduction than the MADSAM patients. Furthermore, a higher frequency of conduction block was observed in the MADSAM patients than in the t-CIDP patients. In contrast, the MADSAM patients exhibited temporal dispersion much less frequently than did the t-CIDP and DADS patients.

**Table 1:** 

	t-CIDP (n = 12)	MADSAM (n = 10)	DADS (n = 3)	
	Mean (±SD), Percent [number]	Mean (±SD), Percent [number]	Mean (±SD), Percent [number]	p Value
Clinical profile				
Age (year)	56 (±12)	56 (±12)	53 (±6)	NS
Male: Female	9∶3	8∶2	2∶1	NS
Disease duration (year)	5.2 (±6.9)	5.4 (±5.4)	3.2 (±1)	NS
Hughes grade scale	2.83 (±0.94)	1.7 (±1.06)	1 (±0)	0.011*, 0.007**, 0.037***
Response to treatment	67% [8/12]	80% [8/10]	100% [3/3]	NS
MRC score				
Total (deltoid+ wrist extensor + iliopsoas + tibialis anterior)	15.7 (±2.6)	18.3 (±1.5)	19.7 (±0.6)	0.015*, 0.013**
CSF protein (mg/dl)	95.3 (±34.7)	55.8 (±31.4)	114.9 (±63.7)	0.002*, 0.028***
CSF IgG index	0.610 (±0.099)	0.560 (±0.134)	0.573 (±0.016)	NS
CSF Q Albumin	0.028 (±0.047)	0.009 (±0.006)	0.014 (±0.011)	NS
Motor conduction study				
Median nerve				
Distal latency (ms)	9.0 (±5.9)	4.7 (±1.0)	14.3 (±12.7)	0.016*, 0.012***
Conduction velocity (m/s)	30.2 (±12.6)	42.3 (±8.3)	41.3 (±0.3)	0.033*
CMAP (mV)	4.6 (±3.4)	6.1 (±3.5)	5.3 (±3.5)	NS
Terminal latency index	0.34 (±0.13)	0.39 (±0.18)	0.18 (±0.11)	NS
Conduction block	58% [7/12]	100% [10/10]	67% [2/3]	0.020*
Temporal dispersion	80% [8/10]	30% [3/10]	100% [3/3]	0.018*, 0.017***

*t-CIDP vs MADSAM, **t-CIDP vs DADS, ***MADSAM vs DADS.

Data are expressed as mean (±SD), median [range] or percent {number}.

t-CIDP, typical chronic inflammatory demyelinating polyradiculoneuropathy; MADSAM, multifocal acquired demyelinating sensory and motor neuropathy; DADS, distal acquired demyelinating symmetric neuropathy, IVIg: Intravenous immunoglobulin, MRC: Medical Research Council, CSF: cerebrospinal fluid, CMAP: compound muscle action potential.

### The sera obtained from the patients with t-CIDP, MADSAM and DADS disrupted the BNB

**Figure 1:**
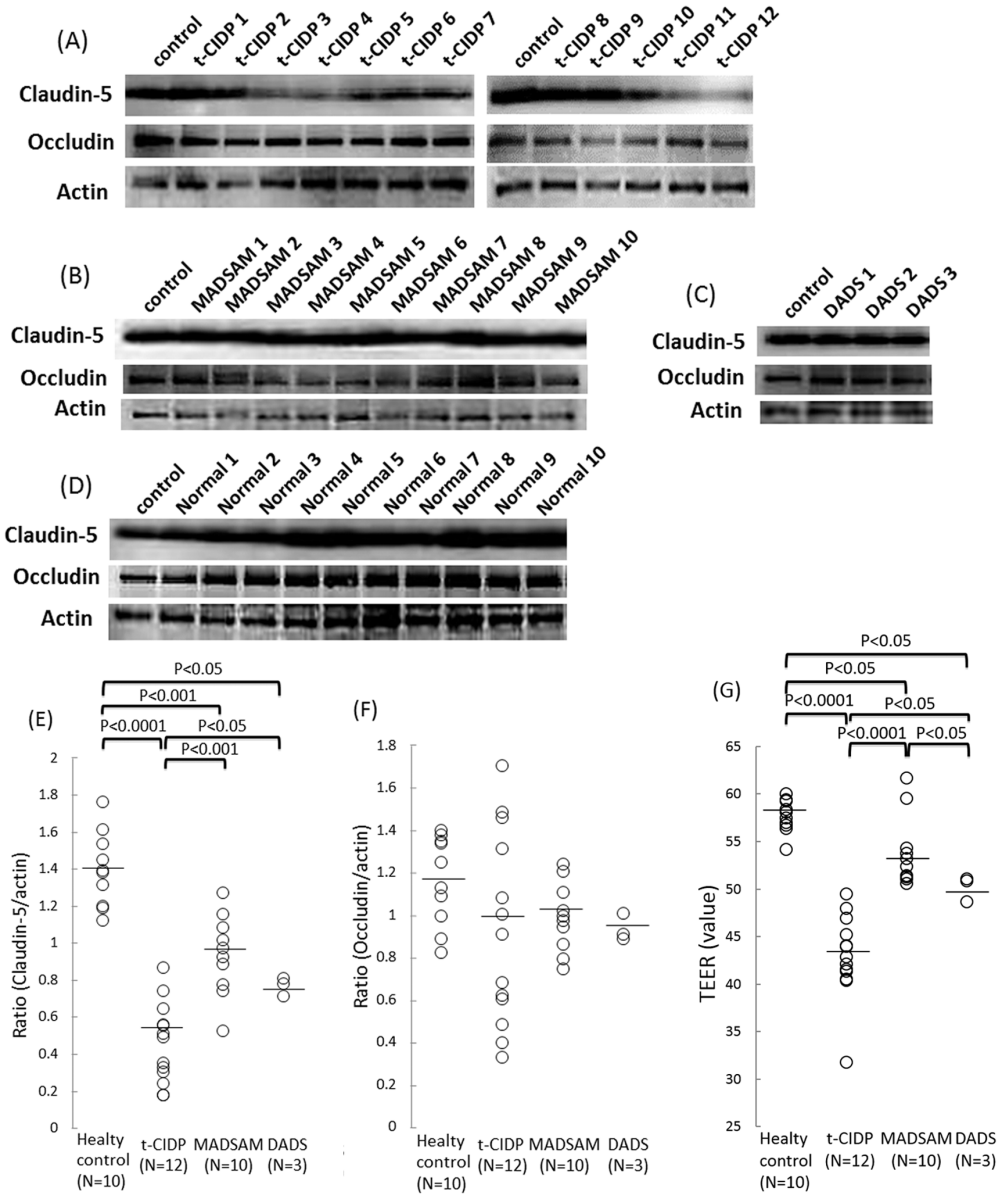
The sera obtained from the patients with t-CIDP, MADSAM and DADS disrupted the BNB. (A) – (D) Effects of the sera obtained from patients with three different phenotypes of chronic inflammatory demyelinating polyneuropathy (CIDP) on the protein levels of claudin-5 and occludin in the FH-BNBs, as determined using a Western blot analysis. The cells were exposed to sera from either patients with typical CIDP (t-CIDP) (A), multifocal acquired demyelinating sensory and motor neuropathy (MADSAM) (B) or distal acquired demyelinating symmetric neuropathy (DADS) (C) or healthy volunteers (D). (E) The sera obtained from the patients with t-CIDP, MADSAM neuropathy and DADS neuropathy decreased the protein ratio of claudin-5 to actin proteins in the FH-BNBs compared to that observed following exposure to the sera from the healthy volunteers. The decrease in the claudin-5 levels in the FH-BNBs was greater after incubation with the sera obtained from the t-CIDP patients than after that with the sera from the patients with MADSAM and DADS. (F) There were no significant differences between the patients with the three different phenotypes of CIDP and the healthy controls regarding the occludin protein levels in the FH-BNBs. (G) The effects of the sera on the transendothelial electrical resistance (TEER) values in the FH-BNBs were also evaluated. Adding sera obtained from the patients with t-CIDP, MADSAM neuropathy or DADS neuropathy resulted in decreased TEER values in the FH-BNBs in comparison with that observed in the cells treated with the sera obtained from the healthy volunteers. Markedly decreased TEER values in FH-BNBs were also observed in the FH-BNBs following incubation with the sera obtained from the t-CIDP patients compared to that noted in the cells incubated with sera from patients with MADSAM or DADS neuropathy. The TEER values were decreased following exposure to the sera obtained from the patients with DADS neuropathy compared to that observed after exposure to the sera obtained from the patients with MADSAM neuropathy. The bars indicate the mean level in each group. Control: non-conditioned DMEM containing 20% FBS. t-CIDP: conditioned medium with 10% sera obtained from patients with t-CIDP diluted with non-conditioned DMEM containing 10% FBS. MADSAM: conditioned medium with 10% sera obtained from patients with MADSAM diluted with non-conditioned DMEM containing 10% FBS. DADS: conditioned medium with 10% sera obtained from patients with DADS diluted with non-conditioned DMEM containing 10% FBS. Normal: conditioned medium with 10% sera obtained from a healthy volunteer diluted with non-conditioned medium of DMEM containing 10% FBS.

We first examined the effects of the sera obtained from the patients with the three clinical subtypes of CIDP on the expression levels of tight junction proteins and the TEER values in the FH-BNBs. Consequently, the protein ratio of claudin-5 to actin proteins was significantly lower in the FH-BNBs exposed to sera from the patients with t-CIDP, MADSAM and DADS than in those incubated with sera from the healthy controls, as determined in a Western blot analysis (**Figs. 1 A–E**). In contrast, the ratio of occludin to actin proteins in the FH-BNBs did not change after a challenge with the sera obtained from the CIDP patients or healthy controls (**Figs. 1A–D, F**). Meanwhile, the TEER values in the FH-BNBs were significantly decreased following exposure to the sera obtained from the t-CIDP, MADSAM and DADS patients in comparison to that observed after exposure to sera of the healthy control (**Fig. 1G**). Furthermore, the ratio of claudin-5 to actin proteins and the TEER values observed after exposure to the sera obtained from t-CIDP patients were significantly lower than those observed after exposure to the sera obtained from the MADSAM and DADS patients (**Figs. 1E, G**). Moreover, the TEER values observed after exposure to the sera obtained from the DADS patients were significantly lower than those observed after exposure to the sera obtained from the MADSAM patients, although the ratio of claudin-5 to actin proteins was not significantly different between the two groups (**Figs. 1E, G**).

### Correlations between the clinical, laboratory and electrophysiological findings and BNB malfunction in the patients with CIDP

**Figure 2:**
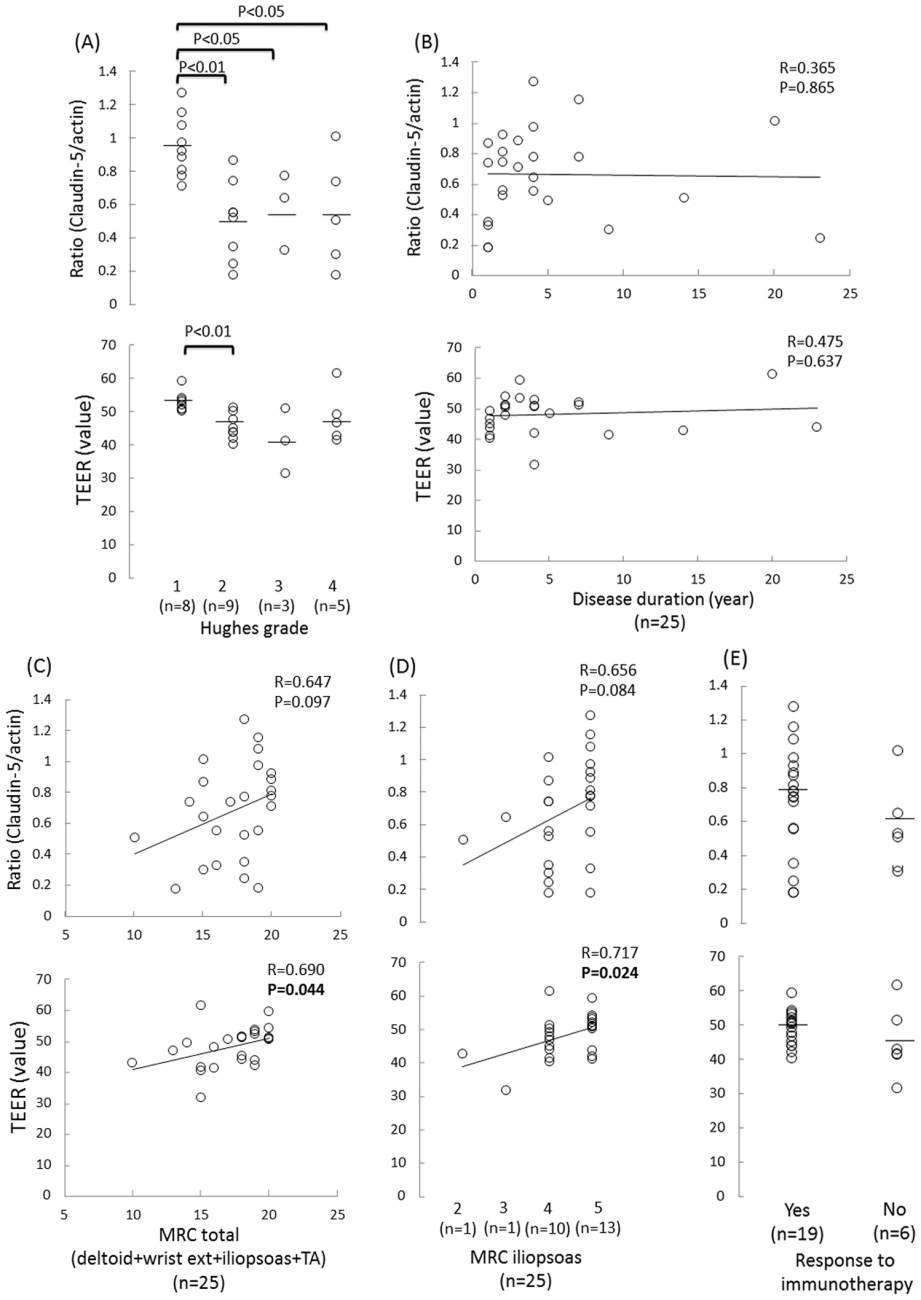
Associations between the clinical findings and BNB malfunction in the patients with CIDP. Correlations between the claudin-5 to actin protein ratios and the TEER values in the FH-BNBs following exposure to sera and the clinical parameters in the patients with CIDP. Associations between the claudin-5 to actin protein ratios and TEER values and the Hughes grade (A), duration of disease from onset (B), total Medical Research Council (MRC) scores for four muscle groups (deltoid, wrist extensor, iliopsoas, and tibialis anterior muscles) (C), MRC score for the iliopsoas muscle (D) and response to treatment, including intravenous immunoglobulin (IVIg) and corticosteroids (E). A lower ratio of claudin-5 to actin proteins was significantly associated with a higher Hughes grade, while a lower TEER value significantly correlated with a higher Hughes grade and lower MRC score.

**Figure 3:**
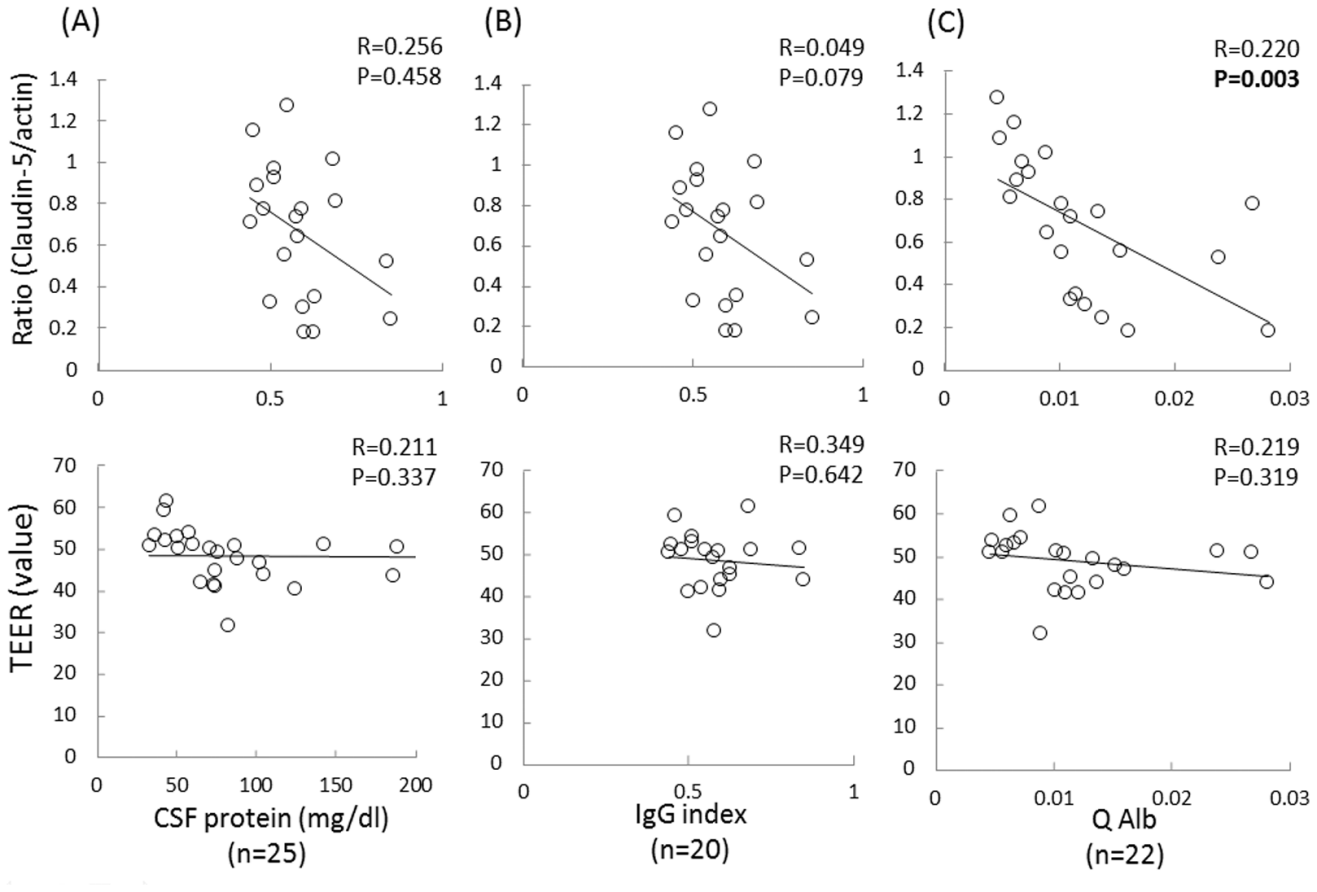
Associations between the CSF parameters and BNB disruption in the patients with CIDP. Correlations between the claudin-5 to actin protein ratios and the TEER values in the FH-BNBs following exposure to sera and the cerebrospinal fluid (CSF) parameters, including the CSF protein level (A), IgG index (B) and albumin ratio (Q Alb) (C) in the patients with CIDP. A lower ratio of claudin-5 to actin proteins was significantly associated with a higher Q Alb.

**Figure 4:**
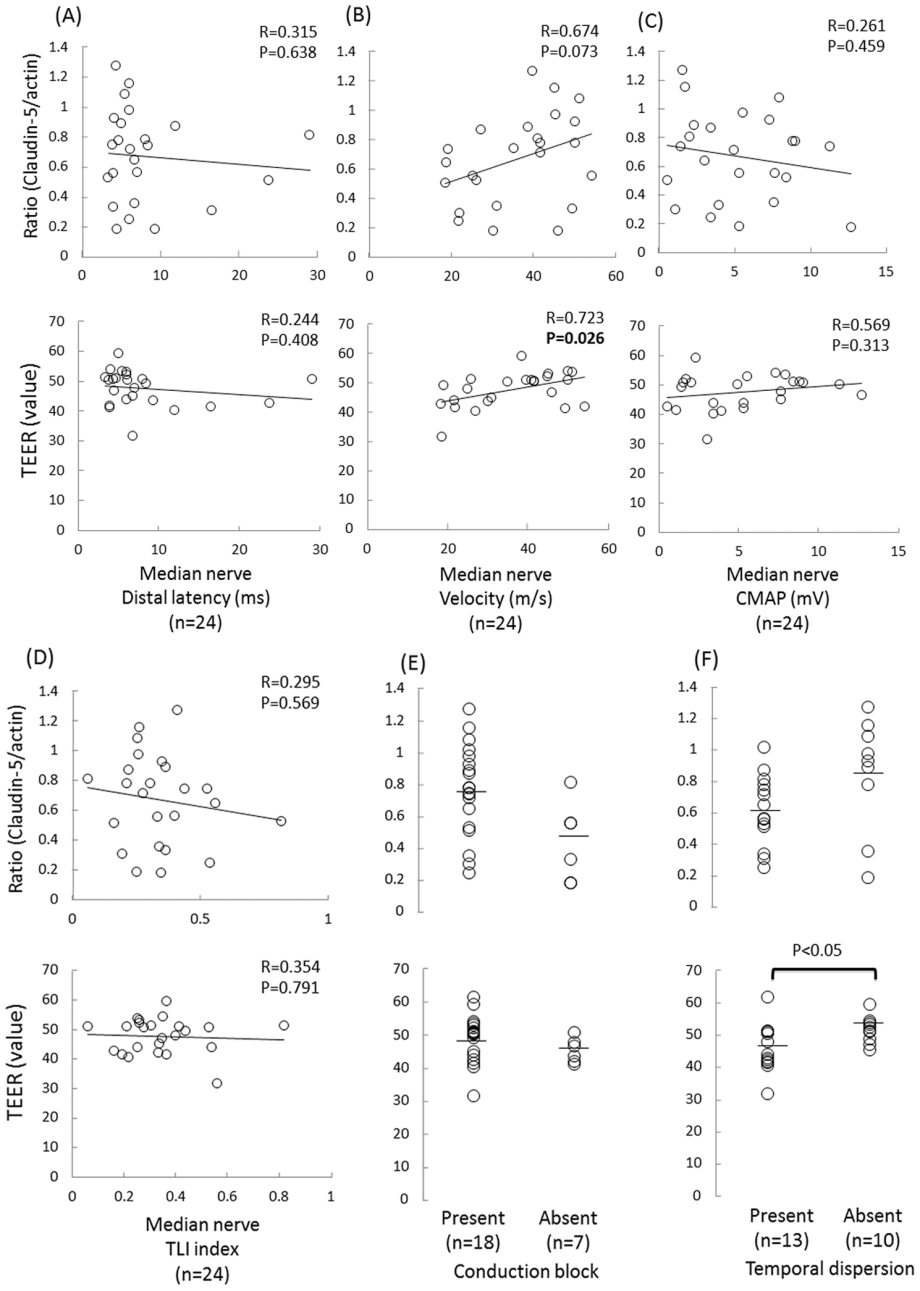
Correlation between the electrophysiological findings and BNB disruption in the patients with CIDP. Associations between the claudin-5 to actin protein ratios and the TEER values in the FH-BNBs following exposure to sera and the electrophysiological findings of the median nerve, including the distal nerve latency (A), conduction velocity (B), compound muscle action potential (CMAP) (C), terminal latency index (TLI index) (D) and presence of conduction block (E) or abnormal temporal dispersion (F) in the patients with CIDP. A lower TEER value was highly associated with slower motor nerve conduction and the presence of abnormal temporal dispersion.

We next examined the associations between the clinical, laboratory and electrophysiological findings and the ratio of claudin-5 to actin proteins and/or the TEER values in the FH-BNBs exposed to the sera from the CIDP patients. Consequently, the decrease in either the claudin-5 protein level or TEER value in the FH-BNBs was found to be associated with the clinical severity. In addition, a lower ratio of claudin-5 to actin proteins significantly correlated with a higher Hughes grade (**Fig. 2A**) and higher Q Alb level (**Fig. 3C**), while a lower TEER value was significantly associated with a higher Hughes grade (**Fig. 2A**), lower MRC score (**Fig. 2C**), particularly in the iliopsoas muscle (**Fig. 2D**), more pronounced slowing of the motor nerve conduction in the median nerve (**Fig. 4B**) and higher frequency of abnormal temporal dispersion (**Fig. 4F**). In contrast, no significant differences were noted between the claudin-5 to actin protein ratio or TEER value and the duration of disease from onset (**Fig. 2B**), response to immunotherapy (**Fig. 2E**), concentration of CSF proteins (**Fig. 3A**), IgG index (**Fig. 3B**), distal latency (**Fig. 4A**), conduction block (Fig. 4E) or CMAP amplitude (**Fig. 4C**) or TLI index (**Fig. 4D**) in the median nerve.

## Discussion

According to the 2010 EFNS/PNS guidelines, CIDP comprises several clinical subtypes, including t-CIDP, MADSAM and DADS, based on the distribution of signs and symptoms [3]. Electrophysiological examinations provide important information regarding the pathogenesis of CIDP, as the distribution patterns of demyelinating lesions differ substantially between the different clinical phenotypes of CIDP [14]. These observations prompted us to hypothesize that differences in the patterns of BNB disruption at least partly determine the distribution of demyelinating lesions and clinical phenotypes of CIDP [10]. In cases of t-CIDP, motor nerve conduction studies frequently show a prolonged distal latency or duration of the distal CMAP, suggesting that demyelination predominantly may affect the distal nerve terminals, where the BNB is most vulnerable, during the initial phase of the disease [10], [14]. However, demyelination also affects the intermediate nerve trunk after a long course of disease in individuals with t-CIDP, due to gradual disruption of the BNB in the nerve trunk. This phenomenon reflects profound slowing of nerve conduction, conduction block and/or abnormal temporal dispersion in the intermediate nerve segments, as identified on motor nerve conduction studies [10]. These disease processes suggest the importance of BNB breakdown in the development of t-CIDP. In contrast, electrophysiology studies of MADSAM have characterized the disease as involving multifocal nerve conduction block in the intermediate nerve trunks, with preservation of the nerve terminals and roots [10], [19], suggesting the presence of multifocal demyelination in these regions. The pattern of BNB disruption appears to differ between MADSAM and t-CIDP, as the multifocal breakdown of the BNB at the site of conduction block may be required for the development of the former condition [10]. The hypothesis suggested by the findings of an electrophysiological studies is of great interest because it may explain the clinical variety of CIDP; however, it is not adequately supported by the results of pathological or cell biological examinations. Only one report regarding pathological changes in the endoneurial microvessels of patients with CIDP has been published to date [11]. This report described the characteristic of pathological changes in tight junction proteins, including a decrease in the level of claudin-5 and altered localization of ZO-1 on sural nerve biopsy samples obtained from t-CIDP patients. However, it remains unclear whether breakdown of the BNB is involved in the pathogenesis of atypical CIDP.

In the present study, we used our previous established human BNB-derived endothelial cells [16] and assessed the degree of BNB damage following exposure to sera by calculating the changes in the protein ratio of claudin-5 to actin proteins and measuring the TEER value [18]. Our results demonstrated that the sera obtained from the patients with three clinical phenotypes of CIDP all significantly decreased claudin-5 expression and the TEER value in the FH-BNBs, suggesting that humoral factors present in the sera of MADSAM and DADS patients, as well as t-CIDP patients, induce the BNB malfunction. The decrease in the claudin-5 protein level and TEER values observed following exposure to the sera obtained from the t-CIDP patients was more remarkable than that observed after incubation with the sera obtained from the patients with MADSAM or DADS. These findings indicate that the severity of BNB breakdown differs depending on the clinical phenotype of CIDP; humoral factors in the sera of t-CIDP patients may cause more severe BNB damage than those present in the sera of patients with MADSAM or DADS. These results partly support the hypothesis suggested by the electrophysiological studies regarding the importance of BNB breakdown induced by humoral factors in t-CIDP sera.

We next examined the associations between the clinical, laboratory or electrophysiological findings and the degree of BNB damage following exposure to the sera obtained from the CIDP patients. Consequently, the severity of BNB damage after exposure to the sera significantly correlated with both a higher Hughes grade and lower MRC score, particularly in the iliopsoas muscle, which reflect the presence of clinical disability and proximal muscle weakness, respectively. Severe BNB breakdown was also found to be associated with a decrease in the speed of conduction in the median nerve in addition to abnormal temporal dispersion, thus indicating the presence of demyelination in the intermediate segments. Furthermore, this damage correlated with an increased Q Alb value, which may reflect disruption of the BNB surrounding the nerve roots. Taken together, these findings suggest that the breakdown of the BNB induced by humoral factors in CIDP sera results in a wide range of symptoms of demyelination from the intermediate nerve trunk to the nerve root, and correlates with both clinical disability and proximal muscle weakness characteristics of t-CIDP. On the other hand, no associations were observed between impairment of the BNB and the duration of the disease or response to immunotherapy in the CIDP patients in our study. This finding suggests that BNB damage does not become more severe as the duration of disease increase, and that the extent of such damage cannot be used to predict the response to treatment.

Katz et al. reported that patients with demyelinating sensory polyneuropathy and distal weakness can be classified as having DADS, in order to distinguish the phenotype from t-CIDP [8]. In addition, two-thirds of patients with DADS have IgM monoclonal gammopathy, and the disease is usually associated with anti-MAG antibodies [20], [21]. DADS associated with positivity for anti-MAG antibodies, termed anti-MAG neuropathy, is separated from CIDP according to the 2010 EFNS/PNS guidelines [3], [22]. In contrast, DADS without anti-MAG antibodies is often considered to be a variant of CIDP, and some reports have described differences in the response to immune treatment between DADS patients with and without anti-MAG antibodies [23], [24]. In the present study, we assessed the effects of sera obtained from three patients with DADS without anti-MAG antibodies, and found that the level of BNB damage after exposure to the sera from these patients was milder than that observed following exposure to the sera of the t-CIDP patients. In addition, we demonstrated a prolonged distal latency and smaller terminal latency index, both of which suggest preferential demyelination in the distal nerve terminals, to be more frequent in the patients with DADS, although these findings did not correlate with the severity of BNB damage. These results suggest that the phenotypic discrepancies observed between t-CIDP and DADS may be due to differences in the location of BNB breakdown; namely, the “DADS phenotype” may be associated with primary involvement at the distal nerve terminal with a vulnerable BNB, as the humoral factors in DADS sera do not induce substantial BNB malfunction at the nerve trunk, compared to that observed in the setting of t-CIDP.

Based on the hypothesis suggested by the finding of electrophysiological studies, the conduction block in the nerve trunk noted in patients with MADSAM is thought to always be accompanied by focal breakdown of the BNB [10]. However, our present results suggest that this conduction block may have little relationship with the involvement of the BNB induced by humoral immunity, as the BNB damage observed after exposure to the sera obtained from the MADSAM patients was milder than that detected after exposure to the sera obtained from the t-CIDP and DADS patients and the presence of conduction block did not correlate with the severity of BNB damage after exposure to sera from any of the patients. Nevertheless, due to the *in vitro* nature of our experiments, we were unable to fully estimate the importance of the BNB breakdown induced by cellular immunity in the MADSAM patients because our data could not be used to elucidate the contribution of the sera to the passage of inflammatory cells across the BNB. It is possible that focal BNB breakdown at site(s) of conduction block is involved in the pathophysiology of MADSAM via the up-regulation of inflammatory cytokines and adhesion molecules. Therefore, further studies to clarify the association between BNB damage and cellular immunity in the setting of MADSAM are required.

The clinical and electrophysiological features of MADSAM and multifocal motor neuropathy (MMN) are very similar, although MADSAM can be distinguished from MMN by the presence of overt sensory involvement, infrequency of anti-GM1 IgM autoantibodies and responsiveness to steroid treatment [6], [25]. In addition, the severity of BNB breakdown appear to differ between the two diseases. We previously reported that the sera derived from MMN patients decrease the claudin-5 protein level and the TEER values in the BNB by approximately 50% compared to that observed in healthy controls based on the same *in vitro* BNB model [18]. Comparing the finding of our previous and present studies, BNB damage is more severe in patients with MMN than in those with MADSAM, suggesting that humoral factors play a greater role in the onset of MMN than in that of MADSAM. This hypothesis implies that the pathological mechanism underlying the development of MMN are significantly different from those of MADSAM, although the two diseases share similar clinical features.

In conclusion, the present findings suggest that the severity of BNB breakdown differs depending on the clinical phenotype of CIDP, and may be associated with both the clinical disability and demyelination in the nerve trunk. Our data imply that measurements of the degree of the BNB breakdown would be useful diagnostic biomarkers for predicting both the clinical phenotype and course of CIDP. However, because this was retrospective, further prospective, large-scale studies are required to validate our findings. The accumulation of further data regarding the molecular mechanism(s) responsible for the BNB impairment observed in patients with CIDP may also lead to the development of improved or novel treatments for CIDP.

### Ethic approval

The study was approved by the ethics committee of Yamaguchi University.

### Provenance and peer review

Not commissioned; externally peer reviewed.

## References

[1] Vallat JM, Sommer C, Magy L (2010) Chronic inflammatory demyelinating polyradiculoneuropathy: diagnostic and therapeutic challenges for a treatable condition. Lancet Neurol 9: 402–412. 20298964 10.1016/S1474-4422(10)70041-7

[2] Hughes R (2010) Chronic Inflammatory Demyelinating Polyradiculoneuropathy. J Clin Immunol 30: S70–73. 20393791 10.1007/s10875-010-9399-0

[3] Van den Bergh PY, Hadden RD, Bouche P, Cornblath DR, Hahn A, et al (2010) European Federation of Neurological Societies/Peripheral Nerve Society guideline on management of chronic inflammatory demyelinating polyradiculoneuropathy: report of a joint task force of the European Federation of Neurological Societies and the Peripheral Nerve Society - first revision. Eur J Neurol 17: 356–363. 20456730 10.1111/j.1468-1331.2009.02930.x

[4] Research criteria for diagnosis of chronic inflammatory demyelinating polyneuropathy (CIDP). Report from an Ad Hoc Subcommittee of the American Academy of Neurology AIDS Task Force. Neurology 41: 617–618. 2027473

[5] Barohn RJ, Kissel JT, Warmolts JR, Mendell JR (1989) Chronic inflammatory demyelinating polyradiculoneuropathy. Clinical characteristics, course, and recommendations for diagnostic criteria. Arch Neurol 46: 878–884. 2757528 10.1001/archneur.1989.00520440064022

[6] Saperstein DS, Amato AA, Wolfe GI, Katz JS, Nations SP, et al (1999) Multifocal acquired demyelinating sensory and motor neuropathy: the Lewis-Sumner syndrome. Muscle Nerve 22: 560–566. 10331353 10.1002/(sici)1097-4598(199905)22:5<560::aid-mus2>3.0.co;2-q

[7] Viala K, Renié L, Maisonobe T, Béhin A, Neil J, et al (2004) Follow-up study and response to treatment in 23 patients with Lewis-Sumner syndrome. Brain 127: 2010–2017. 15289267 10.1093/brain/awh222

[8] Katz JS, Saperstein DS, Gronseth G, Amato AA, Barohn RJ (2000) Distal acquired demyelinating symmetric neuropathy. Neurology 54: 615–620. 10680792 10.1212/wnl.54.3.615

[9] Saperstein DS, Katz JS, Amato AA, Barohn RJ (2001) Clinical spectrum of chronic acquired demyelinating polyneuropathies. Muscle Nerve 24: 311–324. 11353415 10.1002/1097-4598(200103)24:3<311::aid-mus1001>3.0.co;2-a

[10] Kuwabara S, Misawa S (2011) Chronic inflammatory demyelinating polyneuropathy: Clinical subtypes and their correlation with electrophysiology. Clin Exp Neuroimmunol 2: 41–48.

[11] Kanda T, Numata Y, Mizusawa H (2004) Chronic inflammatory demyelinating polyneuropathy: decreased claudin-5 and relocated ZO-1. J Neurol Neurosurg Psychiatry 75: 765–769. 15090575 10.1136/jnnp.2003.025692 PMC1763546

[12] Kanda T (2013) Biology of the blood-nerve barrier and its alteration in immune mediated neuropathies. J Neurol Neurosurg Psychiatry 84: 208–212. 23243216 10.1136/jnnp-2012-302312

[13] Ubogu EE (2013) The molecular and biophysical characterization of the human blood-nerve barrier: current concepts. J Vasc Res 50: 289–303. 23839247 10.1159/000353293 PMC4030640

[14] Kuwabara S, Ogawara K, Misawa S, Mori M, Hattori T (2002) Distribution patterns of demyelination correlate with clinical profiles in chronic inflammatory demyelinating polyneuropathy. J Neurol Neurosurg Psychiatry 72: 37–42. 11784822 10.1136/jnnp.72.1.37 PMC1737682

[15] Kuwabara S, Misawa S, Mori M, Tamura N, Kubota M (2006) Long term prognosis of chronic inflammatory demyelinating polyneuropathy: a five year follow up of 38 cases. J Neurol Neurosurg Psychiatry 77: 66–70. 16361595 10.1136/jnnp.2005.065441 PMC2117396

[16] Abe M, Sano Y, Maeda T, Shimizu F, Kashiwamura Y, et al (2012) Establishment and characterization of human peripheral nerve microvascular endothelial cell lines: a new in vitro blood-nerve barrier (BNB) model. Cell Struct Funct 37: 89–100. 22672995 10.1247/csf.11042

[17] Hughes RA, Newsom-Davis JM, Perkin GD, Pierce JM (1978) Controlled trial prednisolone in acute polyneuropathy. Lancet 2: 750–753. 80682 10.1016/s0140-6736(78)92644-2

[18] Shimizu F, Omoto M, Sano Y, Mastui N, Miyashiro A, et al (2014) Sera from patients with multifocal motor neuropathy disrupt the blood-nerve barrier. J Neurol Neurosurg Psychiatry 85: 526–537. 23926278 10.1136/jnnp-2013-305405

[19] Magda P, Latov N, Brannagan TH 3rd, Goldfarb A, Chin RL, et al (2005) Multifocal acquired sensory and motor neuropathy: electrodiagnostic features. J Clin Neuromuscul Dis 7: 10–18. 19078776 10.1097/01.cnd.0000176973.53512.e4

[20] Van den Berg L, Hays AP, Nobile-Orazio E, Kinsella LJ, Manfredini E, et al (1996) Anti-MAG and anti-SGPG antibodies in neuropathy. Muscle Nerve 19: 637–643. 8618562 10.1002/(SICI)1097-4598(199605)19:5<637::AID-MUS12>3.0.CO;2-K

[21] Chassande B, Léger JM, Younes-Chennoufi AB, Bengoufa D, Maisonobe T, et al (1998) Peripheral neuropathy associated with IgM monoclonal gammopathy: correlations between M-protein antibody activity and clinical/electrophysiological features in 40 cases. Muscle Nerve 21: 55–62. 9427224 10.1002/(sici)1097-4598(199801)21:1<55::aid-mus8>3.0.co;2-f

[22] Hadden RD, Nobile-Orazio E, Sommer C, Hahn A, Illa I, et al (2006) European Federation of Neurological Societies/Peripheral Nerve Society guideline on management of paraproteinaemic demyelinating neuropathies: report of a joint task force of the European Federation of Neurological Societies and the Peripheral Nerve Society. Eur J Neurol 13: 809–818. 16879290 10.1111/j.1468-1331.2006.01467.x

[23] Larue S, Bombelli F, Viala K, Neil J, Maisonobe T, et al (2011) Non-anti-MAG DADS neuropathy as a variant of CIDP: clinical, electrophysiological, laboratory features and response to treatment in 10 cases. Eur J Neurol 18: 899–905. 21199182 10.1111/j.1468-1331.2010.03312.x

[24] Mygland A, Monstad P (2003) Chronic acquired demyelinating symmetric polyneuropathy classified by pattern of weakness. Arch Neurol 2003 60: 260–264. 10.1001/archneur.60.2.260 12580713

[25] Katz JS, Saperstein DS (2001) Asymmetric Acquired Demyelinating Polyneuropathies: MMN and MADSAM. Curr Treat Options Neurol 3: 119–125. 11180748 10.1007/s11940-001-0046-1

